# Design of Injectable Nanocomposite Hydrogels for Controlled Release of Nanoparticles

**DOI:** 10.1021/acsabm.5c00821

**Published:** 2025-07-15

**Authors:** Wilhelm R. Glomm, Erlend Sørlie, Sabina P. Strand, Le T. Truong, Andreas K. O. Åslund

**Affiliations:** † Department of Biotechnology and Nanomedicine, SINTEF Industry, Sem Sælands Vei 2a, N-7034 Trondheim, Norway; ‡ Department of Chemical Engineering, Norwegian University of Science and Technology, Sem Sælands vei 4, N-7034 Trondheim, Norway

**Keywords:** nanocomposite hydrogels, drug delivery, injectable hydrogels, principal component analysis, release kinetics

## Abstract

Nanocomposite hydrogels, *i*.*e*.; a combination of hydrogels and nanoparticles, have emerged as a promising platform for drug delivery owing to the high degree of design flexibility. Despite the wealth of literature on nanocomposite hydrogels, there is scant experimental data on the release of drug-loaded nanoparticles from hydrogels. Here, we report on the incorporation and subsequent release of dye-loaded nanoparticles from alginate-based hydrogels. We investigated the rheological properties of alginate and alginate-poly­(ethylene oxide) hydrogels as a function of nanoparticle loading, as well as the time-dependent nanoparticle release profiles. The effects of hydrogel compositional parameters and nanoparticle loading on viscosity and release profiles were investigated using principal component analysis (PCA) and partial least-squares regression (PLSR), respectively. Additionally, the release profiles were fit using Zero and First-order models as well as the Higuchi, Hixson–Crowell and Korsmeyer–Peppas kinetic models for drug release. We found that the effect of nanoparticle concentration on gel viscosity is strongly dependent on the alginate concentration, with nanoparticles contributing to the gel structure at higher polymer concentrations. Release of nanoparticles from the hydrogels was well fit by a modified first-order kinetic model, comprising a significant burst followed by a concentration-dependent release of nanoparticles. Collectively, our findings provide insight into how hydrogels can be used as reservoirs for controlled delivery of nanoparticles.

## Graphical abstract

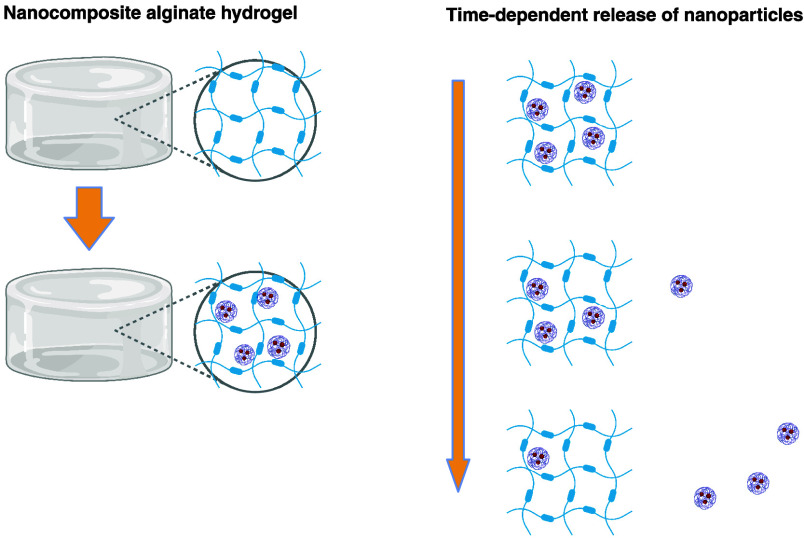

## Introduction

Hydrogels – *i*.*e*.; three-dimensional hydrophilic polymer networks which can absorb and retain large amounts of water or similar biological fluids – are widely used in biomedical applications such as drug delivery, tissue engineering and wound healing (see *e*.*g*. reviews from Peers^1^ et al., Thang^2^ et al. and Vasile^3^ et al.). Their highly porous structure allows for loading and sustained release of hydrophilic drugs by controlling the swelling behavior. The release kinetics can be further modulated by tailoring the composition and characteristics of the hydrogel, including polymer type, molecular weight and cross-linking density. While the high amount of water in hydrogels allows for excellent biocompatibility, it also imparts some limitations, such as poor loading of hydrophobic drugs and a high initial (burst) drug release. To address these issues, nanocomposite hydrogels, *i*.*e*.; a combination of hydrogels and nanoparticles, have emerged as a promising platform for drug delivery.^4–11^

Inclusion of nanoparticles in the hydrogel systems provides a high degree of design flexibility as each of the components can be altered independently. This in turn offers several advantages including tunable drug release, multiple drug loading (*e*.*g*. hydrophilic and hydrophobic), increased stability and protection of sensitive drugs from degradation in biological fluids.^4–6^ The incorporated nanoparticles can also impart stimuli-responsiveness to the nanocomposite, whereby the therapeutic can be released on-demand by use of an external trigger. One application of nanocomposite hydrogels involves the use of nanoparticles as the primary release barrier, with the hydrogel acting both as a secondary release barrier and a scaffold for localizing the nanoparticles at the site of administration, *e*.*g*. at the site of injection.^5,9^ In the case of injectable nanocomposite hydrogels, using nanoparticle fillers is also known to modulate the mechanical properties, including stiffness, adhesiveness, and self-healing (see *e*.*g*. review by Piantanida^10^ and references therein). Injectable hydrogels can be categorized as either *in situ*-forming or shear-thinning hydrogels^7^ and should behave like a fluid during injection and then jellify as quickly as possible after ejection to avoid undesirable spreading in the surrounding tissue. Nanoparticle fillers can be used to modulate or increase the rate of gel formation or self-healing by acting as dynamic, multivalent cross-linkers.^10^

Despite the wealth of literature on nanocomposite hydrogels as drug delivery systems, there are scant experimental data on the use of hydrogels as a release barrier for drug-loaded nanoparticles. In this embodiment of nanocomposite hydrogels, the hydrogel acts as a depot and primary release barrier for nanoparticles which in turn only release their cargo upon reaching the desired location, either via bioaccumulation or stimuli-response. While there are reviews mentioning the possibility of release of nanoparticles from nanocomposite hydrogels (see *e*.*g*. Jiang^4^ and references therein), to the best of our knowledge there are no reports describing the release profiles of nanoparticles. Thus, there is a lack of systematic studies on how nanocomposite hydrogel composition and nanoparticle loading affect the release of nanoparticles. Here, we have investigated the effect of hydrogel composition and nanoparticle loading on the viscosity and release profile of nanoparticles using an alginate-based hydrogel and polystyrene nanoparticles as model particles. Sodium alginate (alginate) was selected as the basis for an electrostatically cross-linked hydrogel owing to its common use in biomedical applications (see *e*.*g*. review by Lee and Mooney^12^). Alginates are water-soluble anionic polysaccharides composed of β-d-mannuronic (M) and α-l-guluronic acid (G) residues. The formation of alginate gels is due to binding of Ca^2+^ or other di- or multivalent cations with G-residues (or blocks), forming egg-box structures.^13^ Gel formation is either considered “external” or “internal” depending on whether the cation–typically Ca^2+^—is added to or gradually released within the alginate solution. External gelation occurs when Ca^2+^ diffuses from the outside to the inside of the alginate solution, e.g. by mixing two solutions. In contrast, internal gelation is based on gradual release of Ca^2+^ within the alginate solution, resulting in uniform distribution of the cations, slower gelling kinetics and thus a structurally homogeneous hydrogel.^14^ A common method to achieve release of Ca^2+^ inside the alginate solution is to use insoluble salts such as calcium carbonate, which becomes soluble at acidic pH. Here, we used d-(+)-Gluconic acid δ-lactone (GDL) as the acidifier, resulting in gradual dissolution of calcium carbonate and thus in Ca^2+^-mediated cross-linking of alginate e.g. as described by Djemaa et al.^15^ Additionally, poly­(ethylene oxide) (PEO) was used as a co-gelator owing to its common use as a humectant.^16^ Fluorescently labeled polystyrene nanoparticles were selected to represent a model nanoparticle-drug combination wherein the drug would not be released into and via the hydrogel, but only be present within the nanoparticles, to allow for monitoring of nanoparticle–rather than model drug–release. The effects of hydrogel compositional parameters and nanoparticle loading on viscosity and release profiles were investigated using principal component analysis (PCA) and partial least-squares regression (PLSR), respectively. To elucidate the mechanism for nanoparticle release, the release profiles were also fit to commonly reported kinetic models.

## Materials and Methods

### Materials

d-(+)-Gluconic acid δ-lactone (GDL) (For GC, ≥99% purity), Poly­(ethylene oxide) (PEO, Mw 100 and 600 kDa) and sodium chloride were purchased from Sigma-Aldrich (Merck–details). Calcium carbonate nanopowder (*d* = 15–40 nm) was purchased from American Elements. Sodium alginate–Protanal LF 10/60 (average molecular weight 89 kDa) was obtained from FMC Biopolymers. Ethylenediaminetetraacetic acid (EDTA) disodium dihydrate (≥99.0% purity) was obtained from G-Biosciences. Fluorophorex 2108B Fluorescent Polystyrene Nanoparticles (mean hydrodynamic diameter 200 nm, excitation 460 nm, emission 500 nm) for release studies were obtained from Phosphorex LLC. Polystyrene nanoparticles for rheology studies (hydrodynamic diameter ∼200 nm) synthesized via emulsion polymerization (see e.g., Arshady^17^) were prepared in-house.

### Preparation of Stock Solutions and Dispersions

Stock solutions of sodium alginate (6 wt %), 100 kDa PEO (10 wt %) and 600 kDa PEO (5 wt %) were prepared in deionized (DI) water and allowed to completely hydrate and solubilize overnight with gentle mixing. A 10 mM stock solution of sodium chloride was prepared and stored for further use. A 1.2 wt % suspension of calcium carbonate nanoparticles was prepared and sonicated in an ice bath for six cycles of 30 s at an amplitude of 70% using a Branson 450 Digital Sonifier.

### Hydrogel Preparation

For all the experiments described here, the weight ratio between calcium carbonate and alginate was kept constant at 0.1, and GDL was added in a 2.5 molar ratio to the calcium carbonate. A typical hydrogel preparation procedure was as follows: Predetermined amounts of the stock solutions/dispersions of alginate, PEO, calcium carbonate nanoparticles and DI water corresponding to the target gel composition were weighed out in a 20 mL glass vial and mixed using a roller mixer for 60 min until homogeneous. Subsequently, 2.5 mL samples of the mixture were transferred to 4 mL glass vials. For nanoparticle-containing samples, a polystyrene suspension was added to the samples according to the intended nanoparticle concentration, and the samples were subsequently diluted to equal volumes with DI water. As a reference sample, a nanoparticle suspension in DI water corresponding to the most concentrated gel sample was used. Four replicates of 0.5 mL from each sample were then transferred to 1.5 mL gas chromatography vials with screw caps. To initiate gelation, 60 μL of a freshly made 60 mg/mL GDL solution was added to each sample, followed by gentle mixing until the mixtures began to thicken. In order to investigate whether gelation had occurred, the vials were turned upside down 5, 10, 20, 30, and 60 min after addition of GDL. If the sample did not flow, it was considered a gel.

### Rheology Measurements

The rheological properties of the hydrogels prepared as described above were characterized at 25 °C using a standard three-step flow/thixotropy test on a rotational Kinexus Lab+ Rheometer (NETZSCH) with rSpace 2.0.0 software. To prevent slippage, serrated parallel plates were used for both the upper (20 mm diameter, PU20, NETZSCH) and lower (40 mm diameter, PL40, NETZSCH) geometries. Measurements were done on freshly cured and aged (20 h) gel samples. To facilitate reproducibility between different instruments, the measurement sequence was as follows:

- Set the temperature to 25 °C, load 0.8 g of gel to the center of the bottom geometry, adjust the gap between the plate geometries to 1 mm and allow the sample to stabilize, ensuring that it is at rest
- Step 1: Apply a low shear rate of 0.1 Hz for 30 s to measure the resting viscosity
- Step 2: Apply a high shear rate of 100 Hz for 30 s to break down the sample structure, simulating an injection
- Step 3: Apply a low shear rate of 0.1 Hz for 10 min to measure sample recovery

Four viscosity values were extracted from each thixotropy measurement, as indicated in Figure 1 below: The initial resting viscosity (μ_0_) at the end of step 1, viscosity after shear (μ_100 Hz_), the initial recovery viscosity (μ_rec_) 15 s into step 3 and the end viscosity (μ_end_) at the end of step 3. The three-step flow test program, with times of the extracted viscosity values indicated, is shown in Figure 1.

**Figure 1:**
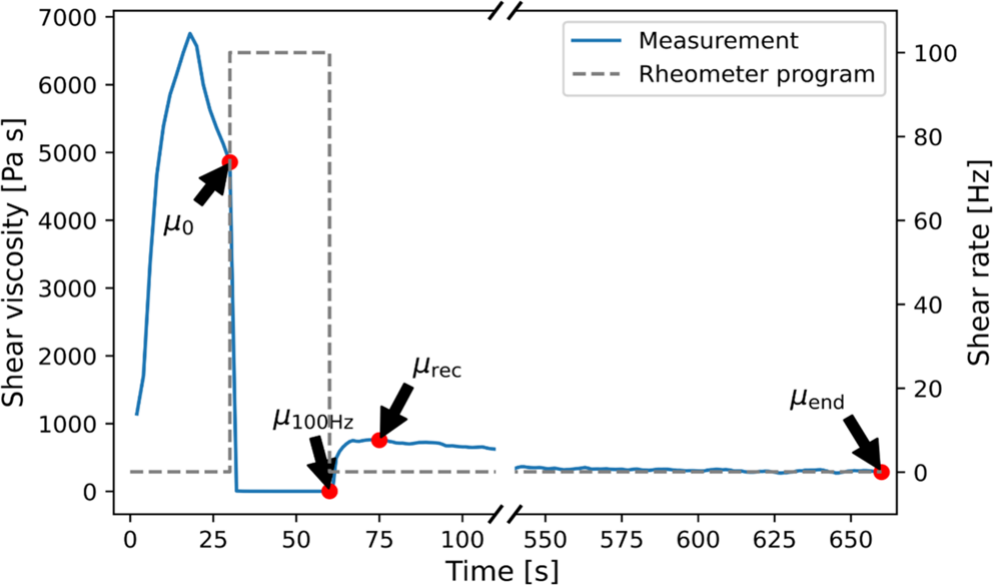
Illustration of the three-step flow curve test used for rheology characterization and a typical example of a measurement after gelling is shown, with selected measurement values indicated by red points and arrows. For visibility, the figure has a broken axis between 100 and 550 s. The initial viscosity (μ_0_), the 100 Hz viscosity (μ_100 Hz_), the initial recovery viscosity (μ_rec_), and the end viscosity (μ_end_) are shown.

### Nanoparticle Release Studies

Nanoparticle-containing hydrogels were prepared as described above and allowed to incubate in a heating cabinet at 37 °C overnight. After incubation, 1 mL of the release medium (10 mM sodium chloride solution) was added on top of each gel replicate sample and the samples were further incubated at 37 °C. At predetermined times, 500 μL of the upper (liquid) phase was carefully pipetted from the vials and transferred to 1.5 mL Eppendorf tubes. The withdrawn sample was immediately replaced with 500 μL of fresh release medium. For the reference sample (positive control), 10 μL of the nanoparticles was diluted with 140 μL release medium in an Eppendorf tube and incubated along with the gel sample at 37 °C.

The fluorescence intensity of the release medium was measured using a BioTek Synergy H1 plate reader using 96-well plates (Costar, 3915). 100 μL of the liquid was added to each well, and the intensity was measured using excitation and emission wavelengths of 460 and 500 nm, respectively. Calibration curves were generated based on a series of known nanoparticle concentrations and measured in the same manner as for the samples of release media, with three replicate measurements per nanoparticle concentration. The fluorescence intensity versus nanoparticle concentration were subsequently fitted by first-order linear regression.

Additionally, dynamic light scattering measurements were performed on the extracted samples in 40 μL cuvettes (ZEN0040, Malvern Panalytical) using a Malvern Zetasizer Advance Pro Blue (Malvern Panalytical) to confirm release of nanoparticles. Here, 40 μL of each extracted sample was added to the cuvette and equilibrated for 1 min at 25 °C before making four consecutive size distribution measurements.

### Dissolution of the Hydrogels

After the release profile measurements were completed, the gels containing Fluorophorex 2108B particles were dissolved to measure the concentration of remaining particles in the gels. All remaining liquid volume was pipetted out of the GC vials, and 1 mL of 2 wt % EDTA solution was added in order to form chelates with calcium, thus dissolving the hydrogels. After addition of EDTA, the GC vials were vortexed, and the samples were examined at different time points up to 1 week after mixing.

### Data Analysis

Principal component analysis (PCA) was performed on a data set comprised of compositional data for the hydrogels and data obtained from the three-step flow curves using the PCA­() function in the scikit-learn library in Python. Outliers were detected and removed from the data set using the function stats.zscore­() from the scipy library in Python. Partial least-squares regression (PLSR) was performed on the nanoparticle release data using various functions from the scikit-learn library in Python. 10% of the data was separated from the rest using the train_test_split­() function to create a validation data set. The rest of the data was used to train the model. Further treatment of the data was performed separately to assess the robustness of the resulting model. The data was standardized across all data columns using the function sklearn.preprocessing.StandardScaler­(), then the optimal number of latent variables was determined by using the GridSearchCV­()-function minimizing the root-mean-square error of prediction (RMSEP). The model was made using PLSRegression­() with the training set, and evaluated by calculating RMSEP, root-mean-square error of calibration (RMSEC), root-mean-square error of cross-validation (RMSECV), the standard deviation of RMSECV (REMSECV_std) and the R^2^ values of both the training and test sets.

## Results and Discussion

### Rheological Properties of the Hydrogels

The overall shape of the profiles from the three-step flow measurements was similar for all the samples studied here, with a typical example shown in Figure 1. Specifically, the viscosity of the sample decreases over time during the application of a constant shear rate in step 1, which is characteristic of shear-thinning and thixotropic materials. This behavior is consistent with previous research on alginate hydrogels, which also exhibit shear-thinning properties due to the alignment and breakdown of internal structures under shear stress.^18^ The hydrogels did not exhibit significant self-healing. Apart from an initial recovery immediately following the high shear rate in step 2, the material did not recover during the recovery period and instead continued to degrade over time. This lack of recovery indicates that the structural deformation experienced during the high-shear step was permanent, which is characteristic of false thixotropy, where the apparent thixotropic behavior is due to irreversible structural changes–likely between cross-linked domains—rather than reversible shear-induced structural reorganization.^19,20^ Despite the absence of self-healing properties, the typical gel viscosity at the end of the recovery phase was found to be comparable to that of soft tissue,^21^ which is important for use of injectable hydrogels in drug delivery.

### Screening of Hydrogel Compositional Parameters

Initially, different compositions were screened to identify a range of alginate-based hydrogel formulations which successfully gelled according to the method described in the experimental section. Specifically, the alginate concentration was varied from 0.25 to 1.5 wt % and different molecular weights of PEO were used while varying the PEO concentration between 1 and 3 wt %. The results are shown in the Supporting Information, Table S1. Gel formulations with 0.25 wt % alginate did not gel. Similarly, alginate concentrations above 1.5 wt % resulted in brittle, heterogeneous gels. Hence, these compositions were not used in other experiments. It was also observed that increasing the alginate concentration (and subsequently the Ca^2+^ concentration) resulted in faster gelling. The formulations containing 600 kDa PEO gelled considerably slower than the corresponding formulations with 100 kDa PEO. As 600 kDa PEO are comprised of longer chains than 100 kDa PEO, it is possible that the slower gelling time is caused by a higher steric hindrance, slowing down the diffusion of GDL and Ca^2+^ in the gel.^22^

### Effect of Base Hydrogel Composition

Viscosity profiles for pure alginate hydrogels and alginate hydrogels with 1 wt % 100 kDa PEO with different alginate concentrations are shown in Figure 2A. Increasing the alginate concentration resulted in increased viscosity, which is in agreement with literature.^18^ It was also observed that the gels with an increased alginate content were harder to the touch. Similarly to the pure alginate hydrogels, increasing the alginate content of gels containing 100 kDa PEO increases the viscosity of the gels. Comparing the pure alginate formulation with those containing 100 kDa PEO, it can be seen that the addition of humectant (PEO) resulted in a softening of the gels (Figure 2A), which is in line with what has been reported in other studies.^16^

**Figure 2:**
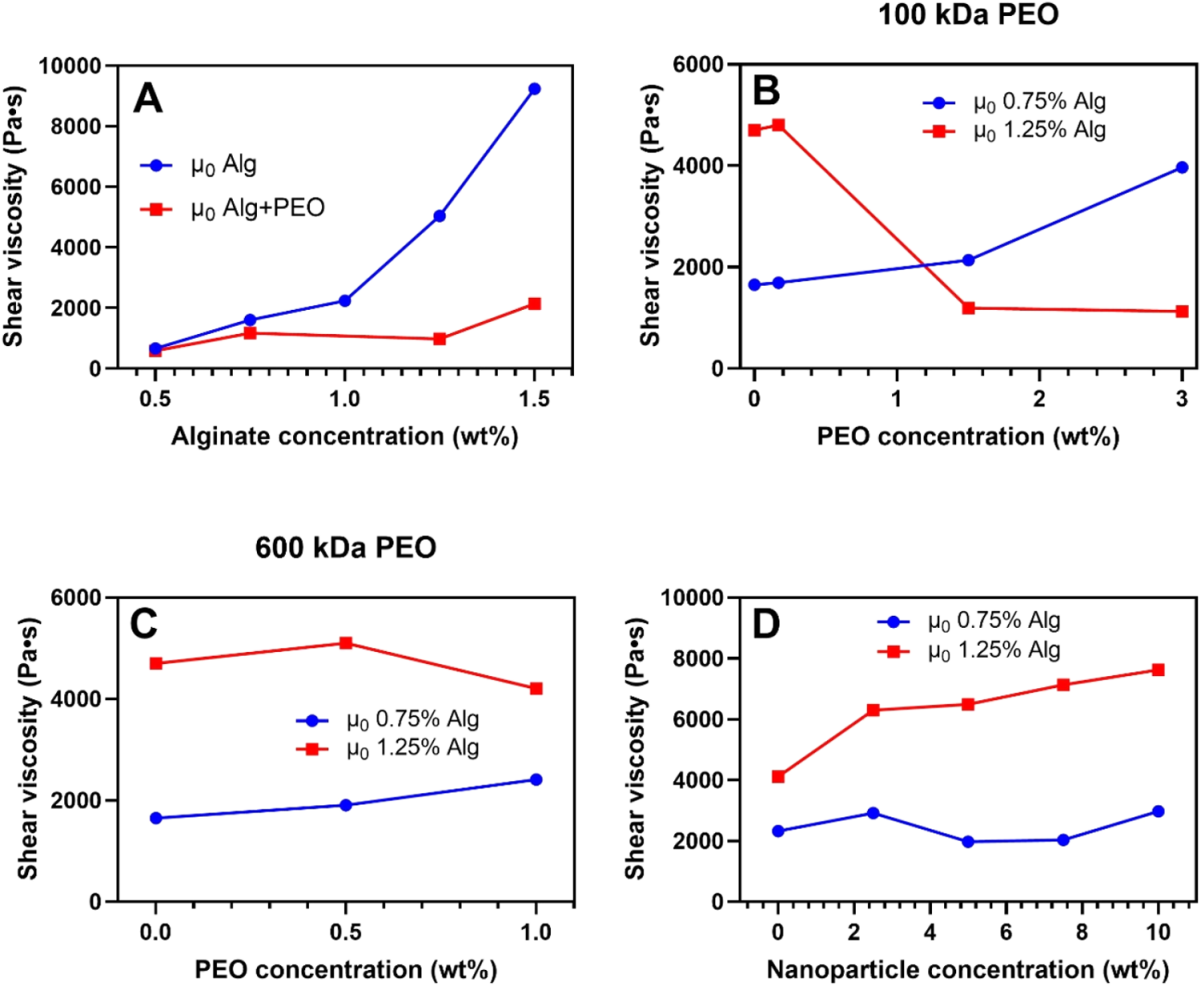
Viscosity measurements (μ_0_) for nanocomposite hydrogels studied here. (A) Hydrogels with varying alginate concentrations (0.5–1.5 wt %) with (Alg + PEO) and without (Alg) 1 wt % 100 kDa PEO. (B–C) Hydrogels with alginate content of 0.75 and 1.25 wt %, and varying concentrations of 100 kDa (B) and 600 kDa (C) PEO. (D) Nanocomposite hydrogels with alginate concentrations of 0.75 and 1.25 wt %, 1 wt % 100 kDa PEO, and varying polystyrene nanoparticle concentrations (0–10 wt %). Measurements were taken 20 h after gelling.

The effect of PEO molecular weight and concentration on the three-step flow curves of alginate-based hydrogels is shown in Figure 2B,2C for 100 and 600 kDa PEO, respectively. For the highest alginate concentration tested (1.25 wt %), addition of >0.25 wt % 100 kDa PEO has a softening effect on the hydrogel, whereas this effect was not observed when using 600 kDa PEO. This suggests that the lower molecular weight PEO might disrupt the alginate gel network, reducing structural integrity. For both molecular weights of PEO investigated, it was seen that for the formulations containing 0.75 wt % alginate, the initial viscosity increased with increasing PEO concentration, signifying that increasing the concentration of PEO also increases the viscosity of the hydrogels. This could also be attributed to an increase in the total amount of gelator/polymer added. Higher concentrations of PEO may contribute to a more robust gel network, potentially enhancing structural integrity. The PEO may occupy the free volume within the hydrogel matrix, increasing the overall density and network connectivity, thereby improving the mechanical properties of the gel.

### Effect of Nanoparticles

Based on the findings for alginate-PEO hydrogels described above, the effect of incorporated nanoparticles on the viscosity of the alginate-based hydrogels was investigated using 0.75 or 1.25 wt % alginate, 1 wt % kDa PEO and varying concentrations (up to 10 wt %) polystyrene nanoparticles as shown in Figure 2D.

The effect of nanoparticle concentration on gel viscosity was found to depend strongly on the alginate concentration (Figure 2D). Specifically, the viscosity of the samples with 0.75 wt % alginate did not exhibit significant changes with varying nanoparticle concentrations, whereas the viscosity of the 1.25 wt % alginate gels increased with higher nanoparticle concentrations. This might suggest that at higher concentrations, nanoparticles contribute to the gel structure, making them more rigid. The absence of this effect at the lower alginate concentration may be due to more free volume for the particles to occupy without disrupting the gel structure, making it necessary to increase the concentration of nanoparticles further to see this effect in those gels. Overall, the results suggest that nanoparticles affect the structure and increase the viscosity of gels at high concentrations. Additionally, it was observed that the gels became increasingly dry and powdery at higher nanoparticle concentrations, indicating that the nanoparticles disrupt the gel network, creating more brittle structures which are overall stiffer, but more prone to breakage. However, some of the observed effects might also be attributed to excess stabilizer from the nanoparticle suspensions.

### Principal Component Analysis (PCA)

To analyze trends across the entire data set obtained from the rheology characterization of the hydrogel samples, the full data set was analyzed using PCA. The explained variance of the principal components from the PCA is shown in Figure 3A.

**Figure 3:**
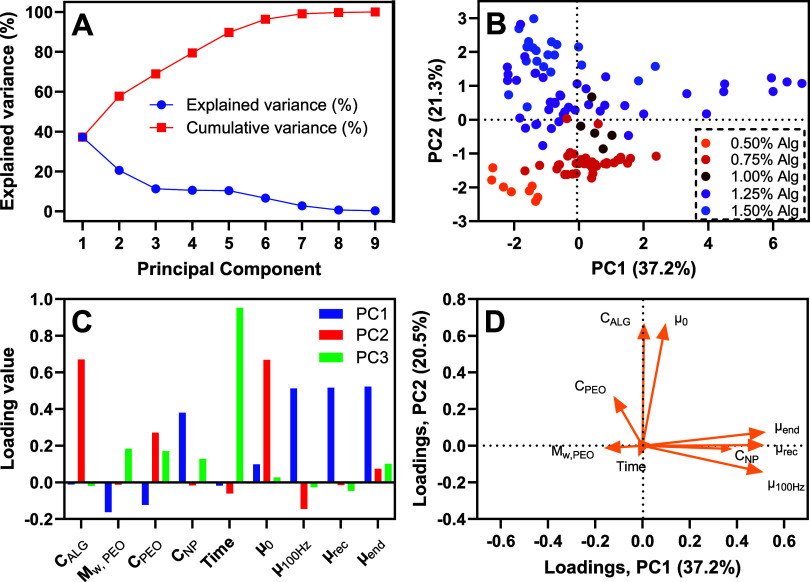
Results from principal component analysis (PCA). (A) Explained Variance of principal components. (B) CA score plot showing the PCA scores for the first two principal components (PC1 and PC2), with data points colored according to the concentration of alginate (*C*_ALG_) in wt %. (C) PCA loadings. The left panel shows the numerical loading values for the first three principal components (PC1, PC2, and PC3) for the alginate concentration (*C*_ALG_), molecular weight of PEO (*M*_w_, PEO), concentration of PEO (*C*_PEO_), nanoparticle concentration (*C*_NP_), time, and the viscosity measurements (μ_0_, μ_100 Hz_, μ_rec_, μ_end_). (D) A biplot showing how the original variables are projected onto PC1 and PC2. The direction and length of the arrows indicate the contribution of each variable to the principal components.

The explained variance of PC1 is 37.2%, which is relatively low compared to typical PCA results for chemical data.^23^ Additionally, the small difference in variance explained by PC1 and PC2 suggests significant measurement noise in the data. A common method for determining the number of PCs to retain is to observe where the explained variance curve flattens out. In this case, the curve levels off after PC3, suggesting that it is optimal to retain three PCs. The goal is usually to retain enough principal components to explain 70–90% of the variance in the data set.^24^ Requiring four principal components to reach a total explained variance of 70% implies that the data has a complex structure where linear combinations cannot fully describe the trends, or that there is considerable noise in the data set.

The score plot, which shows the projection of the original data points onto the principal component space, used to look for clusters in the data is presented in Figure 3B.

Using alginate concentration as a categorical variable resulted in clear groupings, indicating that alginate concentration was the primary factor influencing the gel structure, whereas other parameters such as time have a weaker effect. Moreover, the clusters for higher nanoparticle concentrations are more spread out along PC1, indicating that addition of nanoparticles significantly affects the gel structure as discussed above. The loadings, which display how each variable contributes to the principal components, are shown in Figure 3C. The tabulated numerical values for the loadings of each PC are shown in Supporting Information (Table S2).

From the loadings of PC1, it can be observed that the initial viscosity (μ_0_) is strongly correlated with the concentrations of alginate (*C*_ALG_) and PEO (*C*_PEO_). This indicates that the viscosity of the hydrogels increases with increasing concentrations of alginate and PEO, which is consistent with viscosity data as presented above. The loadings of PC2 show that the viscosities during (μ_100 Hz_) and after (μ_rec_, μ_end_) the high shear step are strongly correlated with each other and with the concentration of nanoparticles (*C*_NP_). It is negatively correlated with the molecular weight (*M*_w_, PEO) and concentration of PEO. The loadings of PC3 mainly consist of the time or age of the gel, without any significant correlations. This indicates that PC3 and lower-order PCs primarily describe experimental variations in the data set.^19^ Overall, the PCA results verified that the trends observed in individual experiments were consistent across all the experiments conducted. The effect of aging on the gel was not observed, likely due to it being too minor to detect amidst the variations present in the data set. This suggests that while the primary trends are robust, more subtle effects could not be identified for this data set.

### Release of Nanoparticles from the Hydrogels

Time-dependent release profiles of polystyrene nanoparticles from alginate-PEO hydrogels are shown in Figure 4 as cumulative fluorescence (A, C, E) and % nanoparticle release (B, D, F).

**Figure 4:**
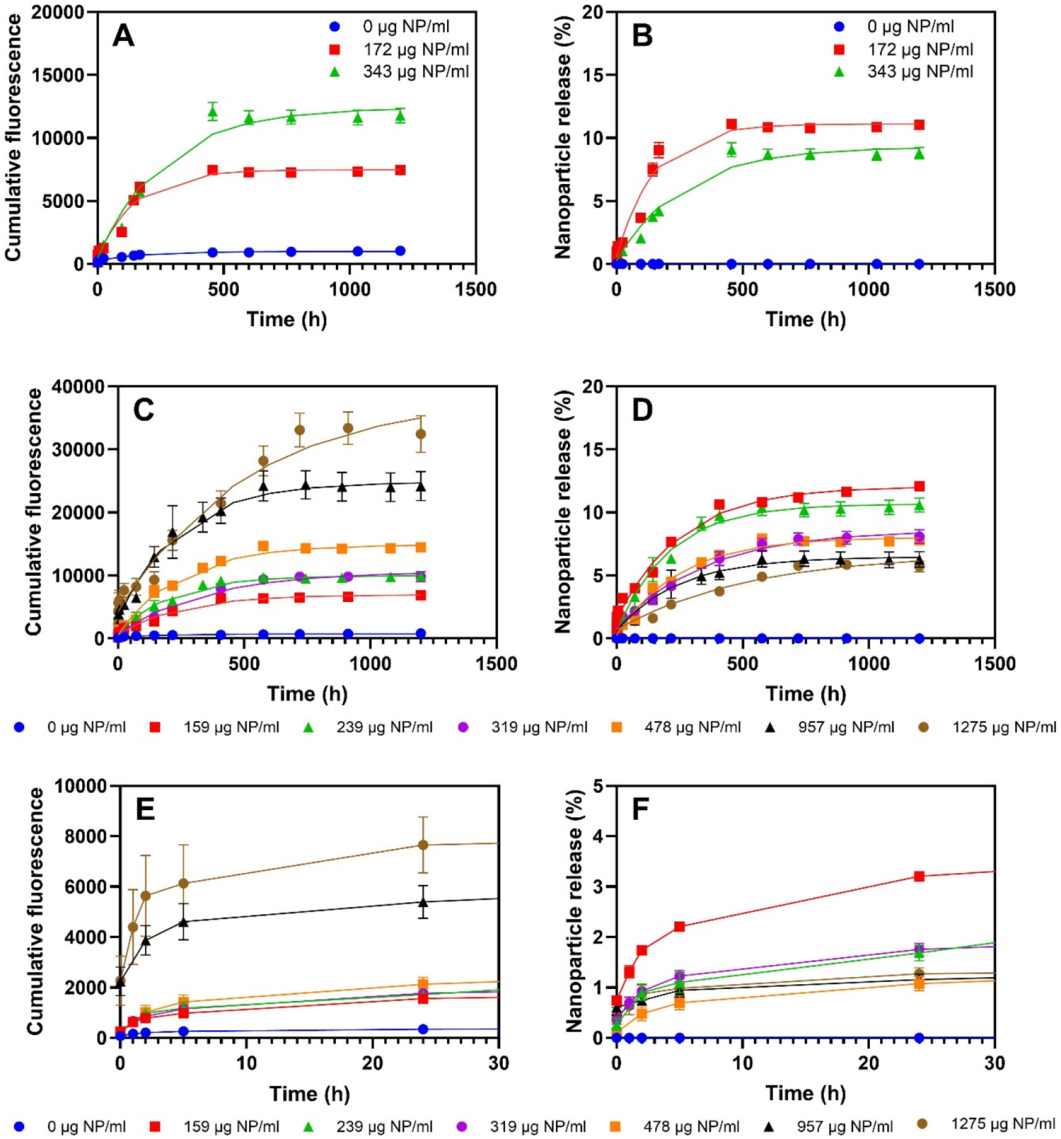
Release profiles of PS nanoparticles from alginate-PEO hydrogels with varying amounts of PS nanoparticles. (A) Cumulative fluorescence intensity of 1.5 wt % alginate hydrogels with 1 wt % PEO. (B) Percentage release of PS nanoparticles from 1.5 wt % alginate hydrogels with 1 wt % PEO. (C) Cumulative fluorescence intensity of 0.75 wt % alginate hydrogels with 1 wt % PEO. (D) Percentage release of PS nanoparticles from 0.75 wt % alginate hydrogels with 1 wt % PEO. (E) First 30 h of cumulative fluorescence intensity of 0.75 wt % alginate hydrogels with 1 wt % PEO. (F) First 30 h of percentage release of PS nanoparticles from 0.75 wt % alginate hydrogels with 1 wt % PEO.

Comparing the cumulative fluorescence and the percentage of released nanoparticles, it is evident that increasing the particle concentration increases the total amount of nanoparticles released, as seen from the cumulative fluorescence intensity from hydrogels with 1.5 wt % alginate (Figure 4A). However, the percentage of nanoparticles released decreases (Figure 4B). This could be caused by the higher particle concentration leading to aggregation within the hydrogel, which could restrict the diffusion pathways and reduce the relative percentage of particles able to escape. Additionally, a higher particle load may result in a denser network of particles within the gel, enhancing interactions between the particles and the gel matrix, thereby hindering their release.^25^

In order to increase the release of nanoparticles, hydrogels with a larger mesh size and more free volume in the structure were prepared by reducing the alginate content to 0.75 wt %. From the release profiles shown in Figure 4C–D, the overall trend for the lower alginate concentration was the same as for 1.5 wt %. Specifically, the overall release was found to be low, and increasing the nanoparticle concentration resulted in a lower percentage of release. Compared to 1.5 wt % alginate gels, 0.75 wt % alginate released a higher percentage of nanoparticles, implying that decreasing the alginate concentration improves the release efficiency. This could be due to the larger mesh size and increased free volume in the hydrogel matrix, which facilitates nanoparticle diffusion. For the highest nanoparticle concentration, a burst release was observed, indicated by a high initial fluorescence compared to the other gels (Figure 4E). This could signify that the gel matrix becomes saturated with nanoparticles, leading to an initial rapid partial release as the gel structure is unable to contain the surplus particles. This was investigated by further increasing the nanoparticle concentration, resulting in noticeable burst release as indicated by the sharp increase in fluorescence intensity (Figure 4E,F).

Due to the low release from the hydrogels, an attempt was made to dissolve the hydrogels to measure the amount of nanoparticles entrapped in the gels. EDTA was used to remove the Ca^2+^ ions from the egg-box structures, thus dissolving the hydrogels.^26^ The attempts at dissolving the gels – even when exposing the samples to EDTA over several days – proved unsuccessful for nanoparticle-containing hydrogels. This might indicate that the nanoparticles interact with the alginate chains, potentially replacing Ca^2+^ and acting as cross-linkers,^27^ which is supported by the observation that the gels with higher nanoparticle content were more difficult to break apart than those with lower nanoparticle content. From the rheology data, release profiles and the dissolution results, we have compiled a schematic overview of the nanocomposite hydrogels as shown in Figure 5. At low alginate and/or nanoparticle concentrations, the gel network is more open, with less interaction between the nanoparticles and the surrounding hydrogel matrix. At high alginate and/or nanoparticle concentrations, the nanoparticles interact strongly with the alginate, effectively acting as cross-linkers. In turn, this affects the time-dependent release profiles both in terms of release rate and the relative population of nanoparticles embedded in the hydrogel network.

**Figure 5:**
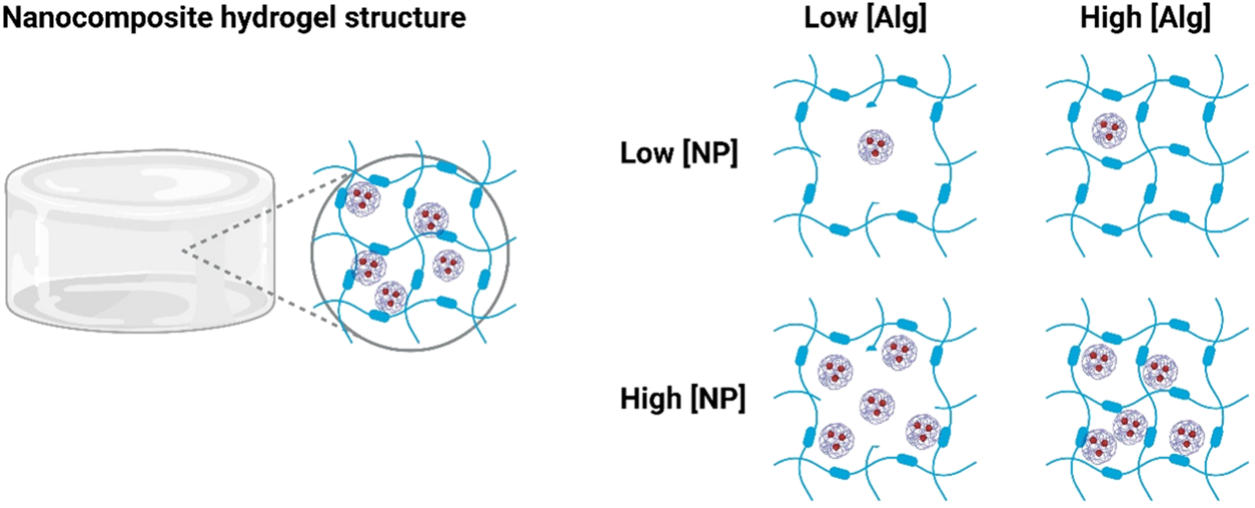
Schematic overview of the nanocomposite hydrogels for low and high concentrations of alginate (Alg) and nanoparticles (NP), respectively. The red dots inside the nanoparticles represent loaded drugs or fluorescent dye.

As the application of nanoparticle-hydrogel composites in research is relatively recent, models for nanoparticle release, or the subsequent drug release from these systems have not yet been developed. Different mass transport phenomena can be involved in the release process, including diffusion of water into the system, drug diffusion out of the system, polymer swelling, erosion, osmotic effects, and others.^28^ Depending on which phenomena are included in the model, its complexity can vary. However, utilizing complex models for systems with many unknown factors necessitates fitting these unknown parameters to experimental data. If too many parameters are fitted simultaneously, the model can become overfitted, causing it to describe the experimental data well but fail to predict new data accurately.^19^ Therefore, we have applied some of the commonly used simple drug release models to elucidate the release mechanisms involved in the release of nanoparticles from the hydrogels.

Diffusional mass transport is almost always involved in drug release (see e.g., review by Siepmann and Siepmann^29^). Drug delivery systems can be classified as having Fickian-based release, meaning the release follows Fick’s laws of diffusion, or non-Fickian release, where the release is more complex and cannot be described by Fick’s laws alone. Non-Fickian release mechanisms involve additional phenomena, the most common being polymer swelling, erosion, and degradation, which influence the drug release kinetics, resulting in behavior that deviates from simple diffusion models.^30^ Diffusion-based drug delivery systems are divided into two classes: reservoir systems and monolithic systems. In reservoir systems, the drugs are encapsulated within a polymer membrane that controls the drug diffusion, which is similar to core–shell nanoparticles. In monolithic systems, the drug is incorporated into a single-block polymer matrix, which is similar to the nanocomposite hydrogels described in this work.

The nanoparticle (NP) release profiles were fit using Zero and First-order release as well as the Higuchi, Hixson–Crowell and Korsmeyer–Peppas kinetic models for drug release. The different methods are summarized as follows:

(i). The Zero-order kinetic model describes a constant release rate independent of drug concentration, such as for drugs with low solubility in water.^30^ For monolithic systems, zero-order release can be observed when release occurs by swelling or erosion of the polymer matrix in a manner that maintains a constant release rate.
1 $$m\mathrm{odel expression}:Q_{t}=Q_{0}+k_{0}t$$
  where *Q_t_* = the cumulative amount of nanoparticle released at time *t*, *Q*_0_ = the amount of nanoparticles released at *t* = 0 and *k*_0_ = zero order model constant (h^–1^). *Q*_0_ is commonly set to zero, but may be nonzero for systems where burst release occurs.
(ii). The first-order kinetic model describes a system where the release rate depends on drug concentration, such as controlled release of hydrophilic drugs from a porous matrix into an aqueous phase.^30^
2 $$Q_{t}=Q_{i}\left(1-e^{-k_{1}t}\right)$$
  where *Q_i_* = the initial amount of nanoparticles inside the system, and *k*_1_ is the first order model constant (h^–1^).
(iii). Equation 2 can be modified by adding *Q*_0_ to account for burst release, leading to a modified first-order kinetic model
3 $$Q_{t}=Q_{0}+Q_{i}\left(1-e^{-k_{1}t}\right)$$
(iv). The Higuchi^31^ kinetic model assumes that Fickian diffusion is the rate-limiting step and the predominant release mechanism.
4 $$m\mathrm{odel expression}:Q_{t}=k_{H}t^{1/2}$$
  where *k*_H_ is the Higuchi release constant.
(v). The Hixson–Crowell^32^ kinetic model is based on the principle that the rate of drug release is related to the change in surface area of the release system as it dissolves, *i*.*e*.; where release occurs through homogeneous erosion or dissolution of spherical particles, and where the dissolution rate is proportional to the surface area.
5 $$m\mathrm{odel expression}:Q_{i}^{1/3}-Q_{t}^{1/3}=k_{HC}t$$
  where *k*_HC_ is the Hixson–Crowell release constant.
(vi). The Korsmeyer–Peppas kinetic model^33^ is a semiempirical power law equation widely used to describe drug release from polymeric systems, and particularly useful for characterizing the drug transport and release mechanisms in systems where the release is controlled by a combination of diffusion and polymer relaxation.

6 $$m\mathrm{odel expression}:\frac{Q_{t}}{Q_{i}}=k_{\mathrm{KP}}t^{n}$$

where *k*_KP_ is the Korsmeyer–Peppas release rate constant and *n* is the release exponent which is used to characterize the transport and release mechanisms considering different structural and geometric characteristics of the system. For cylindrical devices such as used in this study, *n* ≤ 0.43 corresponds to Fickian diffusion in a nonswellable matrix, while *n* = 0.85 corresponds to Case II transport (polymer swelling or zero order release), whereas 0.43 < *n* < 0.85 corresponds to a combination of diffusion and relaxation processes (anomalous transport).

The outcome of applying these mathematical models to the release curves obtained here, as represented by the regression coefficient (*R* ^2^), is shown in Table 1. In order to best compare the models, only the first 60% of the release curves were used for the analysis as recommended for the Korsmeyer–Peppas^33^ model. For all systems, the modified first-order kinetic model (eq 3) gave the best fit for release of nanoparticles from the alginate-based hydrogels studied here. This indicates that there is a significant burst followed by a concentration-dependent release of nanoparticles, which is in agreement with the overall findings as shown in Figure 4. Overall, the Zero-, first-order and Higuchi models were found to fit the release curves comparatively well (Table 1), indicating that Fickian diffusion has a significant contribution to the release mechanism for the systems studied here. The poor fit of the release curves to the Hixson–Crowell model is expected, as this model assumes that the drug is distributed in spherical particles and ignores release from a combination of diffusion and swelling, as well as for heterogeneous erosion. Still, the model was included for comparison owing to its widespread use in modeling of drug release.

**Table 1:** Kinetic Models for Release of Nanoparticles from Alginate-based Hydrogels Expressed by the Values of the Regression Coefficient (*R* ^2^)­^a^

[alginate] (%)	[NP] (μg/mL)	zero-order	first-order	modified first-order	Higuchi	Hixson–Crowell	Korsmeyer–Peppas
1.50	172	0.9417	0.9394	0.9765*	0.8830	0.9359	0.7231
1.50	343	0.9760	0.9757	0.9801*	0.9361	0.9704	0.7970
0.75	159	0.9416	0.9445	0.9882*	0.9638	0.9062	0.8757
0.75	239	0.9637	0.9660	0.9930*	0.9848	0.9550	0.8679
0.75	319	0.9422	0.9443	0.9914*	0.9778	0.9085	0.8972
0.75	478	0.9561	0.9565	0.9897*	0.9306	0.9524	0.7822
0.75	957	0.9791	0.9789	0.9894*	0.8981	0.9550	0.7135
0.75	1275	0.9512	0.9523	0.9791*	0.9259	0.9088	0.7510

a The best fit for each system is marked in bold*.

### Partial Least Squares Regression (PLSR)

To further elucidate release of nanoparticles from the nanocomposite hydrogels, partial least-squares regression (PLSR) was used to fit the data set collected here, using the release as a dependent variable. Results from PLSR performed on nanoparticle release from alginate hydrogels are summarized in Table 2. The optimal number of latent variables was determined to be 2 based on the grid-search. Time and nanoparticle concentration were found to be the parameters with most influence on the release profiles, with nanoparticle concentration being negatively correlated with release, which is in agreement with the release curves in Figure 4. Alginate concentration was determined to be the least influential of the investigated parameters by more than 1 order of magnitude. From Table 2, there is a slight negative correlation between nanoparticle release (in wt %) and alginate concentration, which is in agreement with the overall results shown in Figure 4. Based on the obtained *R* ^2^ values (Table 2), the obtained PLSR model captures the relationships between the independent and dependent variables, but the predictive power is low, possibly caused by insufficient data set size and/or large variations in the data set. The RMSEC values are around 0.5, which is quite high, as values close to 0 are considered to be optimal, and that the maximum value for RMSEC in a normalized data set such as this is 1. The RMSECV values are in the same range as the RMSEC values, indicating that the model is not overfitting the data. Moreover, RMSEC ≤ RMSECV, indicating that the model is quite robust. Ideally, RMSEC should be lower than RMSEP, but this was found to be inconsistent across runs, indicating that there are variations/outliers in the data set, making it so that the test/training split has a somewhat large effect on how well the data fits. This effect can also be seen on the RMSECV_std, which varies significantly across runs. The RMSEP < RMSEC cases are caused by random splits, where the test set has less variation/outliers than the training set. Most likely, this could be removed by including more data, which would also likely result in a better overall fit.

**Table 2:** Results from Partial Least Squares Regression (PLSR) of Release Data for the Parameters Time, Alginate Concentration (*C*_Alg_) and Nanoparticle Concentration (*C*_NPs_)­^a^

run#	time	*C*_Alg_	*C*_NPs_	*R*^2^ (train)	*R*^2^ (test)	RMSEP	RMSEC	RMSECV	RMSECV_std
1	0.807	–0.02	–0.26	0.724	0.74	0.48	0.525	0.532	0.042
2	0.806	–0.01	–0.27	0.719	0.77	0.49	0.531	0.538	0.038
3	0.820	–0.04	–0.28	0.736	0.62	0.58	0.514	0.518	0.044
4	0.811	–0.05	–0.29	0.729	0.70	0.56	0.521	0.525	0.041
5	0.824	–0.01	–0.26	0.735	0.64	0.58	0.515	0.519	0.041
6	0.824	–0.02	–0.25	0.742	0.53	0.63	0.508	0.511	0.036
7	0.816	–0.04	–0.26	0.734	0.65	0.60	0.516	0.523	0.039
8	0.819	–0.03	–0.27	0.722	0.75	0.54	0.527	0.532	0.035
9	0.813	–0.04	–0.29	0.718	0.79	0.50	0.531	0.538	0.025
10	0.813	–0.05	–0.29	0.728	0.71	0.52	0.522	0.524	0.040
Avg	0.815	–0.03	–0.27	0.729	0.69	0.55	0.521	0.526	0.038
Std.	0.007	0.01	0.01	0.008	0.08	0.05	0.008	0.008	0.005

a The correlation coefficient (*R* ^2^) is given for the training (train) and test sets, respectively. RMSECV_std is the standard deviation of the root mean square error of cross-validation (RMSECV).

## Conclusions

Alginate hydrogels, with and without PEO, can be loaded with nanoparticles and may potentially serve as an injectable depot for nanoparticle release. The viscoelastic properties of the nanocomposite hydrogels are a complex interplay of alginate concentration, molecular weight and concentration of PEO and the load of nanoparticles. The addition of PEO to alginate gels can either soften or strengthen alginate hydrogels, depending on the alginate concentration and PEO molecular weight. The hydrogels in this study were able to accommodate up to 10% w/w of the model polystyrene nanoparticles. The release profiles showed an initial burst followed by a concentration-dependent release of nanoparticles, in agreement with the modified first order kinetic model. The overall release of nanoparticles was inversely proportional to nanoparticle concentration and remained low. This shows that the alginate gels act as a significant release barrier for the nanoparticles used in this study. The design of nanocomposite system for controlled release of nanoparticles will require tailoring of interactions between the gel matrix and the nanoparticle properties.

## Supplementary Material



## Notes

Additional data and details on gelation properties as a function of compositional parameters, tabulated loadings from principal component analysis (PDF)

## References

[1] Peers S., Montembault A., Ladavière C. (2020). Chitosan Hydrogels for Sustained
Drug Delivery. J. Controlled Release.

[2] Thang N. H., Chien T. B., Cuong D. X. (2023). Polymer-Based
Hydrogels Applied in
Drug Delivery: An Overview. Gels.

[3] Vasile C., Pamfil D., Stoleru E., Baican M. (2020). New Developments in
Medical Applications of Hybrid Hydrogels Containing Natural Polymers. Molecules.

[4] Jiang Y., Krishnan N., Heo J., Fang R. H., Zhang L. (2020). Nanoparticle–Hydrogel
Superstructures for Biomedical Applications. J. Controlled Release.

[5] Hsu X.-L., Wu L.-C., Hsieh J.-Y., Huang Y.-Y. (2021). Nanoparticle-Hydrogel
Composite Drug Delivery System for Potential Ocular Applications. Polymers.

[6] Ahmad N., Bukhari S. N. A., Hussain M. A., Ejaz H., Munir M. U., Amjad M. W. (2024). Nanoparticles Incorporated Hydrogels for Delivery of
Antimicrobial Agents: Developments and Trends. RSC Adv..

[7] Mellati A., Hasanzadeh E., Gholipourmalekabadi M., Enderami S. E. (2021). Injectable Nanocomposite
Hydrogels as an Emerging Platform for Biomedical Applications: A Review. Mater. Sci. Eng., C.

[8] Ho E., Deng Y., Akbar D., Da K., Létourneau M., Morshead C. M., Chatenet D., Shoichet M. S. (2023). Tunable Surface
Charge Enables the Electrostatic Adsorption-Controlled Release of
Neuroprotective Peptides from a Hydrogel–Nanoparticle Drug
Delivery System. ACS Appl. Mater. Interfaces.

[9] Stanwick J. C., Baumann M. D., Shoichet M. S. (2012). Enhanced Neurotrophin-3 Bioactivity
and Release from a Nanoparticle-Loaded Composite Hydrogel. J. Controlled Release.

[10] Piantanida E., Alonci G., Bertucci A., De Cola L. (2019). Design of Nanocomposite
Injectable Hydrogels for Minimally Invasive Surgery. Acc. Chem. Res..

[11] Oliva N., Conde J., Wang K., Artzi N. (2017). Designing Hydrogels
for On-Demand Therapy. Acc. Chem. Res..

[12] Lee K. Y., Mooney D. J. (2012). Alginate:
Properties and Biomedical Applications. Prog.
Polym. Sci..

[13] Grant G. T., Morris E. R., Rees D. A., Smith P. J. C., Thom D. (1973). Biological
Interactions between Polysaccharides and Divalent Cations: The Egg-box
Model. FEBS Lett..

[14] Draget K. I., Østgaard K., Smidsrød O. (1990). Homogeneous Alginate Gels: A Technical
Approach. Carbohydr. Polym..

[15] Djemaa I. B., Boulmedais F., Auguste S., Tarnowska M., Andrieux S., Drenckhan-Andreatta W. (2024). Glucono-Delta-Lactone-Induced
Alginate
Gelation: New Insights into the Effect of the Cross-Linker Carrier
Type on the Hydrogel Mechanics. Langmuir.

[16] Kapanya A., Somsunan R., Phasayavan W., Molloy R., Jiranusornkul S. (2021). Effect of
Molecular Weight of Poly­(Ethylene Glycol) as Humectant in Interpenetrating
Polymer Network Hydrogels Based on Poly­(Sodium AMPS) and Gelatin for
Wound Dressing Applications. Int. J. Polym.
Mater. Polym. Biomater..

[17] Arshady R. (1992). Suspension,
Emulsion, and Dispersion Polymerization: A Methodological Survey. Colloid Polym. Sci..

[18] Cuomo F., Cofelice M., Lopez F. (2019). Rheological
Characterization of Hydrogels
from Alginate-Based Nanodispersion. Polymers.

[19] Morrison, F. A. Understanding Rheology. Buch; Oxford University Press: New York, NY, 2001.

[20] Mezger, T. G. The Rheology Handbook: For Users of Rotational and Oscillatory Rheometers, 5th revised ed.; Vincentz Network: Hannover, 2020.

[21] Kumar V., Denis M., Gregory A., Bayat M., Mehrmohammadi M., Fazzio R., Fatemi M., Alizad A. (2018). Viscoelastic Parameters
as Discriminators of Breast Masses: Initial Human Study Results. PLoS One.

[22] Lavrentev F. V., Shilovskikh V. V., Alabusheva V. S., Yurova V. Yu., Nikitina A. A., Ulasevich S. A., Skorb E. V. (2023). Diffusion-Limited Processes in Hydrogels
with Chosen Applications from Drug Delivery to Electronic Components. Molecules.

[23] Jolliffe, I. T. Principal Component Analysis. In Springer Series in Statistics, 2ndnd ed.; Springer: New York, 2002.

[24] Greenacre M., Groenen P. J. F., Hastie T., D’Enza A. I., Markos A., Tuzhilina E. (2022). Principal Component Analysis. Nat. Rev. Methods Primers.

[25] Dannert C., Stokke B. T., Dias R. S. (2019). Nanoparticle-Hydrogel
Composites:
From Molecular Interactions to Macroscopic Behavior. Polymers.

[26] Brown L., Green C. L., Jones N., Stewart J. J., Fraser S., Howell K., Xu Y., Hill C. G., Wiwi C. A., White W. I., O’Brien P. J., Litwin V. (2015). Recommendations for
the Evaluation of Specimen Stability for Flow Cytometric Testing during
Drug Development. J. Immunol. Methods.

[27] Thoniyot P., Tan M. J., Karim A. A., Young D. J., Loh X. J. (2015). Nanoparticle–Hydrogel
Composites: Concept, Design, and Applications of These Promising,
Multi-Functional Materials. Adv. Sci..

[28] Chen M. H., Wang L. L., Chung J. J., Kim Y.-H., Atluri P., Burdick J. A. (2017). Methods To Assess Shear-Thinning Hydrogels for Application
As Injectable Biomaterials. ACS Biomater. Sci.
Eng..

[29] Siepmann J., Siepmann F. (2012). Modeling of Diffusion
Controlled Drug Delivery. J. Controlled Release.

[30] Bandyopadhyay, S. Fabrication and Application of Nanomaterials; McGraw-Hill: New York, Chicago, San Francisco, 2019.

[31] Higuchi T. (1963). Mechanism
of Sustained-action Medication. Theoretical Analysis of Rate of Release
of Solid Drugs Dispersed in Solid Matrices. J. Pharm. Sci..

[32] Hixson A. W., Crowell J. H. (1931). Dependence of Reaction
Velocity upon Surface and Agitation. Ind. Eng.
Chem..

[33] Korsmeyer R. W., Gurny R., Doelker E., Buri P., Peppas N. A. (1983). Mechanisms
of Solute Release from Porous Hydrophilic Polymers. Int. J. Pharm..

