# Supplementary material for: Design of Injectable Nanocomposite Hydrogels for Controlled Release of Nanoparticles
Source: ACS Appl Bio Mater. 2025 Jul 15;8(8):7095–104. doi: 10.1021/acsabm.5c00821 (PMC12365918; doi:10.1021/acsabm.5c00821)
Supplement: Supplementary file 1 [file mt5c00821_si_001.pdf]

Supporting information

Design of injectable nanocomposite hydrogels for controlled release of nanoparticles

Wilhelm R. Glomm,[1a] Erlend Sørlie,[2a] Sabina P. Strand,[1] Le T. Truong,[1] Andreas K.O. Åslund[1*]*

[1]Department of Biotechnology and Nanomedicine, SINTEF Industry, Sem Sælands Vei 2a, N-7034 Trondheim, Norway

[2]Department of Chemical Engineering, Norwegian University of Science and Technology, Sem Sælands vei 4, N-7034 Trondheim, Norway

[a]These authors contributed equally to this work

*Corresponding authors: andreas.aaslund@sintef.no wilhelm.glomm@sintef.no

The supporting information is comprised of two tables containing additional data on gelation properties as a function of compositional parameters, as well as details on the loadings from principal component analysis.

Supplementary tables

Table S1: Overview of gelation as a function of compositional parameters for alginate-based hydrogels. The alginate concentration (C_{alg}) varied from 0.25 to 1.5 wt%, and PEO concentration (C_{PEO}) ranged from 1 to 3 wt%, using PEO with different molecular weights ($M_{w,PEO}$) as indicated.

C_{alg} (wt%)	$M_{w,PEO}$ (kDa)	C_{PEO} (wt%)	Gel time
0.25	100	1	Did not gel
0.25	600	1	Did not gel
0.5	100	1	< 1h
0.5	100	1	< 20 min
0.5	600	2	< 24h
0.5	600	3	< 24h
0.75	100	1	< 30 min
0.75	100	1	< 30 min
1.25	100	1	< 10 min
1.25	100	3	< 10 min
1.25	600	1	< 30 min
1.5	100	1	< 5 min
1.5	100	3	< 5 min
1.5	600	1	< 10 min

Table S2: Loadings of each variable on the principal components from the principal component analysis.

Original variable	PC1	PC2	PC3	PC4	PC5	PC6	PC7	PC8	PC9
C_{ALG}	0.0067	0.6701	-0.0197	-0.1543	-0.2309	-0.0120	0.6200	-0.2922	0.0585
$M_{w,PEO}$	-0.1629	-0.0127	0.1832	0.8622	-0.4299	0.0424	0.0513	-0.0388	0.0748
C_{PEO}	-0.1228	0.2713	0.1709	0.3538	0.8125	-0.2923	0.0929	-0.0012	0.0526
C_{NP}	0.3807	-0.017	0.1288	0.1094	0.262	0.8388	0.1431	0.0022	0.1831
Time	-0.0172	-0.0608	0.952	-0.2566	-0.0987	-0.08	-0.0385	-0.0587	0.0522
μ_0	0.0982	0.6685	0.0271	0.0185	-0.0939	0.1218	-0.7163	0.0656	-0.0366
μ_{100Hz}	0.5129	-0.1441	-0.0263	0.1177	0.0383	-0.1936	-0.1633	-0.7807	-0.1629
μ_{rec}	0.5166	0.0031	-0.0463	0.0244	-0.1005	-0.3588	0.0134	0.2755	0.7179
μ_{end}	0.5229	0.0743	0.1014	0.1225	-0.0548	-0.1471	0.2058	0.4691	-0.6393
